# Hemi-Masquelet technique and nailing in a circumferential bone defect of 7 cm after open femoral shaft fracture. A case report^☆^

**DOI:** 10.1016/j.tcr.2024.101066

**Published:** 2024-06-05

**Authors:** Joffrey Boucly, André-Pierre Uzel

**Affiliations:** Université des Antilles, Department of Orthopedic Surgery, Guadeloupe University Hospital Center, 97139 Les Abymes, Guadeloupe

**Keywords:** Masquelet's technique, Open femoral fracture, Bone loss, Induced membrane technique

## Abstract

The treatment of Gustilo-Anderson type III open femoral fracture with large segmental bone defect remains a challenge for orthopedic trauma surgeons. The aims of management are first to prevent the risk of infection and then to reconstruct the bone loss with correct alignment and length. The induced membrane technique (or Masquelet technique) was initially described for tibia nonunion but became over the years an established procedure to treat any kind of large bone defect. The case of a 22-year old male who sustained an open femoral shaft fracture with a circumferential 7-cm bone defect after a car accident is presented. Given the critical size of the bone loss, we chose to manage this patient using a modified-Masquelet technique, in which we stabilized the fracture by an intramedullary femoral nail and filled only the lateral side of the defect with a cement spacer. He went on to have a full and successful union of his fracture 16-weeks after the second stage surgery. The final functional outcomes were excellent allowing the patient to resume all activities without restriction.

## Introduction

The treatment of open femoral fracture with large segmental bone defect remains challenging for surgeons. The aims of management are to prevent the risk of infection and to reconstruct the defect in order to obtain an aseptic union with correct alignment and length of the lower limb and good functional outcomes.

The treatment strategy usually involves two steps: first it consists of irrigation and debridement of the wounds [1], followed then by bone defect reconstruction. Several techniques have been described, including bone transport with distraction osteogenesis (BTDO) [2], a free vascularized fibular bone graft [3] and the induced membrane technique (IMT) or Masquelet technique.

IMT is a two-stage procedure, used to treat large defects up to 25 cm [4]. The first stage involves soft-tissue and bone debridement and fitting an antibiotic-free poly-methylmethacrylate (PMMA) cement spacer into the resultant defect. The spacer's role is to maintain the original length, to prevent fibrous ingrowth into the defect and to induce the development of a surrounding vascularized pseudo-synovial membrane. After 6–8 weeks, the second stage consists of removing the cement and filling the empty area with cancellous bone graft.

We present a case a 7-cm circumferential femoral shaft bone defect secondary to a Gustilo-Anderson type IIIA open fracture treated by a modified-Masquelet technique.

## Case presentation

**Fig. 1:**
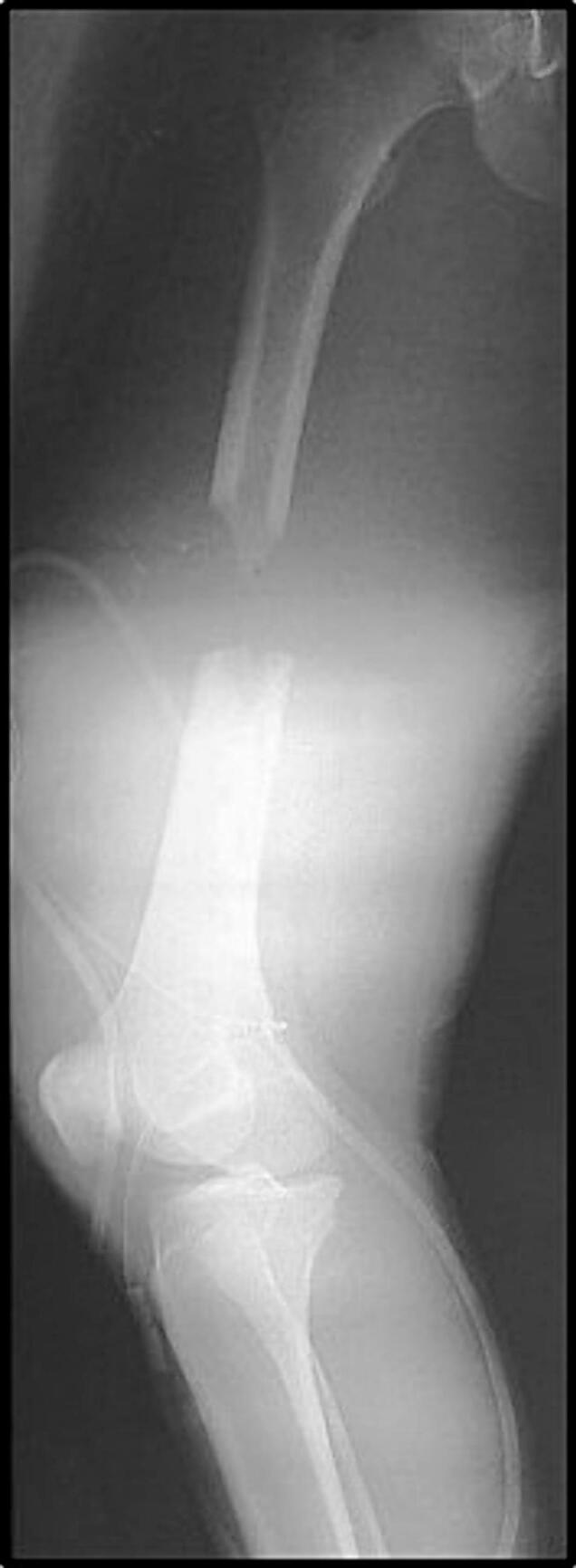
Radiograph showing the circumferential 7-cm defect after Gustilo-Anderson type IIIA femoral shaft open fracture in a 22-year old male patient.

We present a case report of a 22-year-old male who was admitted to our department for a polytrauma after a car accident. His major injury was a Gustilo-Anderson type IIIA open femoral mid-shaft fracture with a 7 cm-long circumferential bone defect (Fig. 1). Associated polytrauma injuries included contralateral distal radius fracture and lateral femoral epicondylar fracture. The usual protocol for open fracture started in the emergency department, including a prophylactic antibiotic treatment (2 g of amoxicillin-clavulanic acid), a tetanus toxoid booster, a sterile dressing of the wounds and an immobilization of the fracture (skin traction). Then the patient was shifted to the operating room for a surgical irrigation, debridement and loose closure of the wounds. The skin traction was left in position and the prophylactic antibiotic treatment was provided for 48 h. A pangonogram was performed a day after surgery to precisely measure the length of the contralateral femur and estimated the size of the bone defect.

**Fig. 2:**
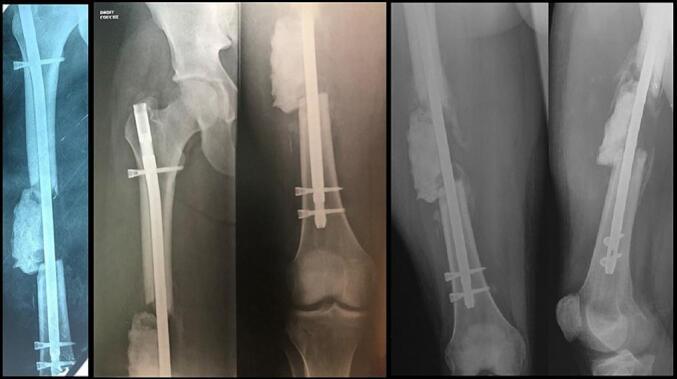
The first stage of Masquelet technique. The stabilization was achieved by an intramedullary nail and a cement spacer was placed into the lateral part of the defect (Left). A spontaneous medial bone regeneration is noticeable on the follow-up views after 8 weeks (middle) and 12 weeks (right).

The first stage of the modified-Masquelet procedure was performed after these 48 h. The patient was placed supine on an orthopedic table, neutral rotational alignment of the hip and the distal femur was controlled by fluoroscopy. Skin traction was applied until having a 7 cm-long area between the two fracture ends and stabilization by a proximal and distal locked intramedullary femoral nail (Dinamic Nail, Citieffe®) was achieved. Then antibiotic-loaded cement (PALACOS® with gentamicin) was placed as a spacer in the lateral part of the defect; medial part of the fracture was left empty (Fig. 2). No weight-bearing was allowed on the limb before 2 months, but the patient immediately started passive mobilization of the hip and the knee with the physiotherapist, in order to prevent stiffening.

**Fig. 3:**
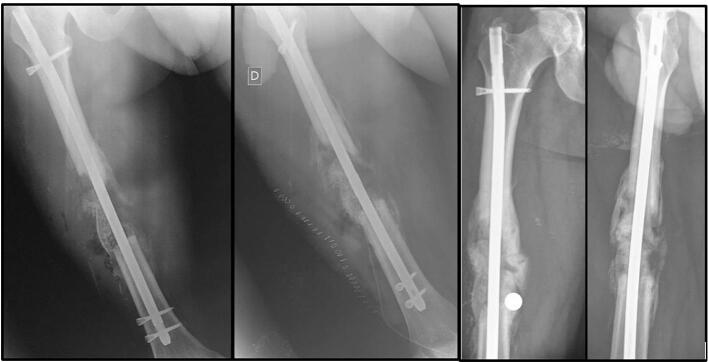
Second stage of modified-Masquelet technique. The cement spacer has been removed and replaced by autologous graft and synthetic bone substitute. No complementary treatment was made to the medial part of the defect. Lateral and antero-posterior X-rays of the femur immediately after surgery (left) and 16 weeks after surgery (right).

After 12 weeks, the second stage of the procedure was done as there was not any sign of infection. Signs of spontaneous medial union were already noticeable on X-rays. The intramedullary femoral nail of the first stage was left in place. We carefully opened the membrane and removed the spacer, and then filled the lateral defect area with bone autograft harvest from an iliac crest (cortico-spongious stitched to nail and spongious bone graft) associated with synthetic bone substitute pellets (MBCP, Biomatlante®) (Fig. 3). The membrane was tightly closed at the end of the surgery. No complementary treatment was made on the medial aspect of the fracture.

**Fig. 4:**
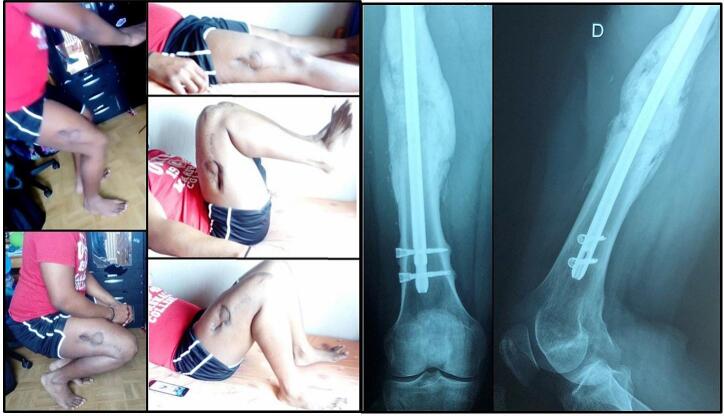
Final clinical (left) and radiological (right) results at 1 year and 2 months follow-up from the initial trauma.

The patient was quickly transferred to a rehabilitation center after surgery. Active and passive mobilization of the hip and the knee were allowed immediately to maintain normal ranges of motion. Partial weight-bearing was started at 8 weeks after the second stage surgery. Full weight-bearing was allowed at 16 weeks when complete bony union with medial and lateral cortical reconstitution was achieved (Fig. 3). The final clinical and radiological result at 1 year and 2 months of the initial trauma show excellent outcome with a complete range of motion of the hip and the knee, no length discrepancy (Fig. 4) and a complete bone remodeling (Fig. 4).

## Discussion

Management of femoral segmental bone defects due to open trauma is challenging. After a first stage of debridement and stabilization of the fracture, it is usually recommended to reconstruct the missing bone [5]. The gold standard is an autogenous cancellous graft if the defect does not exceed the diameter of the bone [6]. When the diaphyseal defect is larger than 6 cm, autologous bone graft alone is not sufficient for healing because of graft resorption, even in good vascularized muscle envelop [7]. Masquelet initially described the IMT as a treatment for tibia nonunion [4]. Over the years, the procedure has been developed for the treatment of significant bone loss in various locations with very good outcomes [8]. The IMT principle includes two stages [6]. The first stage consists of a debridement, a stabilization of the fracture and the placement of a PMMA cement spacer into the defect, in order to induce the formation of foreign-body membrane. After 6–8 weeks, the second stage involves opening the membrane, removing the spacer and filling the empty space with graft. The surrounding membrane then maintains the graft, prevents its resorption and releases inductive factors stimulating osteogenesis [9]. Several changes in the technique has been made with satisfactory resultants, concerning the stabilization device, the type of graft or the composition of the cement (with or without antibiotics). In our case, we stabilize the fracture with a femoral shaft intramedullary nail. We used an antibiotic-loaded cement (PALACOS® with gentamicin) to model the spacer that we introduced only into the lateral aspect of the defect in order to facilitate its removal during the second stage. Removal of a medial hemi-spacer would be difficult and would induce injury of the membrane and soft tissue. We used only bone autograft harvest from an iliac crest (cortico-spongious and spongious bone graft) and synthetic bone substitute pellets, whose osteoconductive role is demonstrated [10,11]. According to Lu et al. [12], the only significant risk factor of post-procedural graft infection is the use of external fixation in the second stage surgery, and graft-infection is one of the risk factors of final nonunion. According to this same review, there are three significant predictive factors of shorter union time, which are the use of antibiotics in the spacer, the use of an all-autologous graft and the use of intramedullary nail as the second stage fixation method. That can explain why complete union was achieved after only 18 weeks after the second-stage surgery in our case.

Even though the induced membrane technique is now relatively popular to treat large femoral shaft defect, several other procedures have been described. Free vascularized fibular grafts have proven to be effective in significant segmental bone loss [3] but require microvascular expertise and can be responsible of serious donor-site morbidities [13]. BTDO is also an effective procedure, using original technique with an Illizarov fixator [2] or modified technique with a single-plan external fixator [14]. However, this procedure requires a long treatment because of slow corticalisation of the newly formed regenerate bone, can be difficult to tolerate and entails non-negligible risks of nonunion at the docking site and fractures of the new formed bone segment after removing external fixation [15,16]. Moreover, this is a stiffening surgery as demonstrated by Pallaro et al. [14], who presented in his series a reduction in knee range of motion in all cases, resulting in poor functional outcomes. In our case report, thanks to the stability of the intramedullary nail, we were able to start physiotherapy quickly and prevent stiffening. An alternative of BTOD by external fixation is the plate-assisted bone segment transport with magnetic intramedullary lengthening nail (PRECICE, NuVasive®), which prevent pin-sites complications and allow early mobilization of the knee and better functional outcomes [17,18]. However, even if results seem promising, this technique requires a long treatment and multiples surgery when the defect is larger than 6–7 cm because of the maximum distraction it can provide for now.

Our technique is a reliable procedure in critical femoral bone defect. According to this case and the literature, the use of intramedullary femoral shaft nail and autologous bone graft optimize the management and reduce the union time, leading to early rehabilitation and excellent functional outcomes.

## Funding

No funds were received in support of this work. No benefits in any forms have been or will be received from a commercial party related directly or indirectly to the subject of this manuscript.

## CRediT authorship contribution statement

**Joffrey Boucly:** Conceptualization, Methodology, Validation, Writing – original draft, Writing – review & editing. **André-Pierre Uzel:** Conceptualization, Data curation, Formal analysis, Methodology, Project administration, Supervision, Resources, Validation.

## Declaration of competing interest

None.
